# Comparative Proteome Analysis Reveals Lipid Metabolism-Related Protein Networks in Response to Rump Fat Mobilization

**DOI:** 10.3390/ijms19092556

**Published:** 2018-08-28

**Authors:** Juanjuan Wang, Mengsi Xu, Xinhua Wang, Jinquan Yang, Lei Gao, Yan Zhang, Xin Huang, Mengli Han, Rui Gao, Shangquan Gan

**Affiliations:** 1 College of Animal Science and Technology, Shihezi University, Shihezi 832000, China; wangjuanjuand10@163.com; 2 State Key Laboratory of Sheep Genetic Improvement and Healthy Production, Xinjiang Academy of Agricultural and Reclamation Sciences, Shihezi 832000, China; xumengsi100@163.com (M.X.); wangxinhua5751@163.com (X.W.); xssxmjq@126.com (J.Y.); w.n007@163.com (L.G.); ahx512@163.com (X.H.); hanmenglimm@163.com (M.H.); 3 Laboratory of Epigenetics, Beijing Institute of Biotechnology, Beijing 100071, China; zany1983@gmail.com; 4 The Key Laboratory of Xinjiang Endemic & Ethnic Diseases and Department of Biochemistry, Shihezi University School of Medicine, Shihezi 832000, China

**Keywords:** altay sheep, rump fat, proteomic, persistent starvation, isobaric tags for relative or absolute quantitation (iTRAQ)

## Abstract

Altay is a typical fat-tailed sheep breed displaying the unique ability to rapidly mobilize fat, which is vital for maintaining a normal metabolism that facilitates its survival in lengthy winter conditions. However, the physiological, biochemical, and molecular mechanisms underlying fat mobilization remain to be elucidated. In this study, the monitoring of rump fat adipocyte sizes disclosed a positive correlation between cell size and fat deposition ability. In addition, we subjected sheep to persistent starvation to imitate the conditions that trigger rump fat mobilization and screened 112 differentially expressed proteins using the isobaric peptide labeling approach. Notably, increased secretion of leptin and adiponectin activated the key fat mobilization signaling pathways under persistent starvation conditions. Furthermore, the upregulation of resistin (RETN), heat-shock protein 72 (HSP72), and complement factor D (CFD) promoted lipolysis, whereas the downregulation of cell death-inducing DFFA-like effector C (CIDEC) inhibited lipid droplet fusion, and the increase in HSP72 and apolipoprotein AI (Apo-AI) levels activated the body’s stress mechanisms. The synergistic actions of the above hormones, genes, and signaling pathways form a molecular network that functions in improving the adaptability of Altay sheep to extreme environments. Our findings provide a reference for elucidating the complex molecular mechanisms underlying rump fat mobilization.

## 1. Introduction

Fat tail is a stress resistance trait in sheep. Some sheep breeds hoard considerable fat in their tails in the forage-rich season, which protects against starvation during lack of forage and cold conditions [1]. Altay sheep are a typical fat-tailed breed, with tail fat representing about a quarter of their carcass weight [2]. These animals are famous for their high meat production and strong adaptability to extreme environments [3]. Fat tail is the result of long-term evolution and natural selection of breeds in a rugged living environment. During warm periods with lush grass growth, adipose tissue is deposited in the rump of Altay sheep. Rump fat undergoes massive decomposition to provide energy for metabolism and resistance to cold weather in periods lacking grass growth, and the tail becomes flat and thin. However, this type of high fat storage ability is associated with a number of problems, in that the economic value of the carcass is reduced (the amount of forage to deposit 1 kg of fat is sufficient to produce 2 kg of meat), and the market share is reduced because of the growing demand for a healthy diet. In Xinjiang, fat-tailed sheep constitute the main breed. The reason for fat deposition in the tail is unclear, and the specific genetic, physiological, and biochemical molecular mechanisms underlying Altay sheep tail fat deposition and mobilization remain to be elucidated.

Fat tissue is not only an important component of the carcass but also participates in many physiological and biochemical responses in the animal body, providing energy for life activities. Adipose tissue is predominantly formed by adipocytes originating from mesenchymal stem cells (MSC). These cells progressively differentiate and eventually form mature adipocytes [4]. The energy storage ability of the adipose tissue (the largest energy storage organ of the body) is related to triglycerides (TG). Adipose tissue can be divided into white adipose tissue (WAT) and brown adipose tissue (BAT). WAT plays an important role in energy balance, storing energy in the form of TG when the body has abundant nutrition. During periods of energy deficiency, WAT is released as free fatty acid (FFA) [5,6]. A small amount of BAT in the body can lead to oxidative decomposition of fat tissue and heat release. Two characteristics of fat deposition have been reported, specifically, the increase in adipocyte number and the enlargement of adipocyte volume. During the embryonic period, adipose tissue development is evident and mainly based on the increase in adipocyte number. After the animal is born, the adipocyte number changes to a limited extent, and fat deposition and synthesis are mainly dependent on the enlargement of adipocyte volumes [7]. Numerous transcription factors, enzyme-encoding genes, and signaling pathways are involved in fat synthesis and decomposition, such as lipoprotein lipase (LPL) [8], CCAAT/enhancer-binding protein (C/EBP) [9], peroxisome proliferator activated receptor (PPAR) [10], fatty acid synthase (FAS) [11], hormone-sensitive lipase (HSL) [12], and adipose triglyceride lipase (ATGL) [13]. In addition, adipocytes secrete a number of cytokines and hormones related to fat metabolism, including adiponectin (APN, ADIPOQ) [14], resistin (RETN) [15], leptin (LEP) [16], visfatin [17], and tumor necrosis factor-α (TNF-α) [18], along with key molecules of signaling pathways, such as peroxisome proliferator-activated receptor (PPAR), AMP-activated protein kinase (AMPK), and mitogen-activated protein kinase (MAPK). However, limited research has been conducted to date on the proteins associated with fat deposition and on mobilization-related genes and signaling pathways.

Isobaric tags for relative or absolute quantitation (iTRAQ) is a high-throughput proteomic technology that allows quantitative comparisons of protein abundance. Until now, limited studies have applied iTRAQ technology to assess proteins in Altay sheep [19]. In the current investigation, we recorded the rump widths and determined serum lipid markers using nine Altay sheep from three age groups (three months old, yearling, and adult) and compared the tail tissue adipocyte sizes of nine Altay sheep from three age groups (young lamb, six months old, and adult), three Wool sheep (adult), and six Altay sheep from “free-feeding” (FF) and “persistent starvation” (PS) groups. In particular, the FF and PS groups could simulate the extreme states of rump fat deposition and mobilization of Altay sheep. Next, we determined the key differentially expressed proteins in the FF and PS groups using iTRAQ technology and the expression patterns of fat synthesis and decomposition genes in the two extreme states. Changes in the expression patterns of the proteins involved in these pathways provide a basis for clarifying the molecular mechanisms underlying rump fat deposition and mobilization in Altay sheep, which may be effectively utilized as a model for human metabolism, nutritional physiology, and diabetes research.

## 2. Results

### 2.1. Persistent Starvation Experiment

To simulate the extreme states of rump fat deposition and mobilization, three Altay adult ewes were provided with limited feed during the winter months in Xinjiang, China. During the gradual reduction in feeding, Altay sheep showed a strong appetite, a good mental status, and slightly reduced weights. In the subsequent four weeks, free drinking water was provided, but no feed. The fold temperatures were maintained between −28 °C and −15 °C. The sheep became anxious but showed no evidence of a pathological state. The foraging desire was extremely strong. After feed deprivation, the average weight was reduced by 11 kg. The rump became loose and soft, with a significant reduction in fat volume.

### 2.2. Changes in Rump Width and Serum Lipid Markers

**Figure 1:**
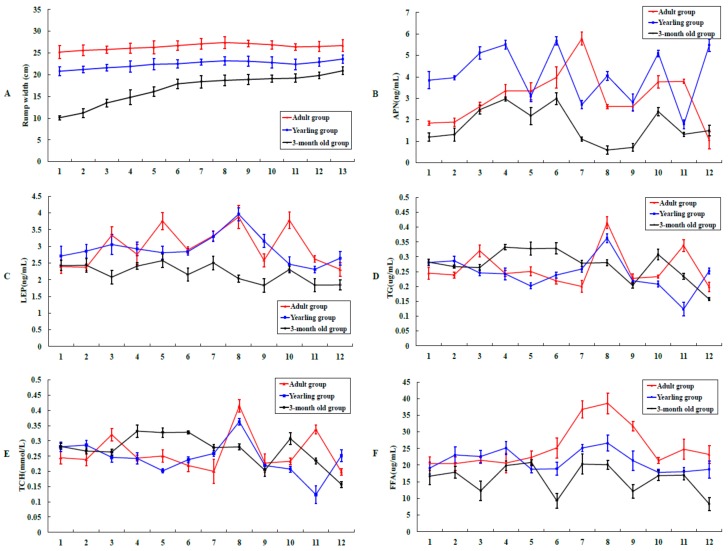
Changes in rump width and lipid marker levels in Altay sheep. (**A**) Rump widths of Altay sheep at three distinct stages of the life cycle recorded monthly from May 2012 to May 2013. (**B**) Serum adiponectin (APN) levels in Altay sheep from the three groups determined from August 2012 to July 2013. (**C**) Serum leptin (LEP) levels in Altay sheep from the three groups determined from August 2012 to July 2013. (**D**) Serum triglycerides (TG) levels in Altay sheep from the three groups determined from August 2012 to July 2013. (**E**) Serum total cholesterol (TCH) levels in Altay sheep from the three groups determined from August 2012 to July 2013. (**F**) Serum free fatty acid (FFA) levels in Altay sheep from the three groups determined from August 2012 to July 2013. The red, blue, and black lines represent the changes in adult, yearling, and three-month-old groups of sheep, respectively. In Figure 1A, the abscissa axis from 1 to 13 represents the months from May 2012 to May 2013. In Figure 1B–F, the abscissa axis from 1 to 12 represents the months from August 2012 to July 2013.

The average rump widths (cm) of Altay sheep in each age group (three months old, yearling, and adult) were monitored. During the course of the year, the rump width changed significantly from 10.1 cm to 20.9 cm, 20.8 cm to 23.6 cm, and 25.2 cm to 27.7 cm, in the three-month-old lamb, yearling, and adult groups, respectively. Under cold winter conditions, the rump width values appeared to show a slight downward trend (Figure 1A). The data suggest that under free-feeding conditions, the rump fat contents in Altay sheep respond to the corresponding environmental conditions, especially in adult sheep. Notably, no decrease in rump width was obvious for the three-month-old group. 

Serum samples were collected from three-month-old, yearlings, and adult Altay sheep for the evaluation of lipid markers. Our results disclosed no seasonal variability in the serum levels of APN (Figure 1B) and LEP (Figure 1C) in Altay sheep. The APN content in adult sheep reached a peak of 5.8 ng/mL in February 2013, and that in yearling and three-month-old sheep peaked in January 2013 at 5.5 ng/mL and 2.9 ng/mL, respectively. The levels in yearling and adult sheep were significantly higher than those in three-month-old sheep. TG (Figure 1D) and total cholesterol (TCH) (Figure 1E) levels in sheep in all groups were not significantly different. However, the FFA levels showed an obvious peak during the long winter period in Xinjiang, China. For example, the FFA concentration in adult sheep increased from 23 μg/mL in January to 37 μg/mL in March and subsequently decreased to 22 μg/mL in May. This phenomenon was more evident in adult sheep than in three-month-old and yearling animals (Figure 1F).

### 2.3. Cell Volumes of Sheep Adipocytes

**Figure 2:**
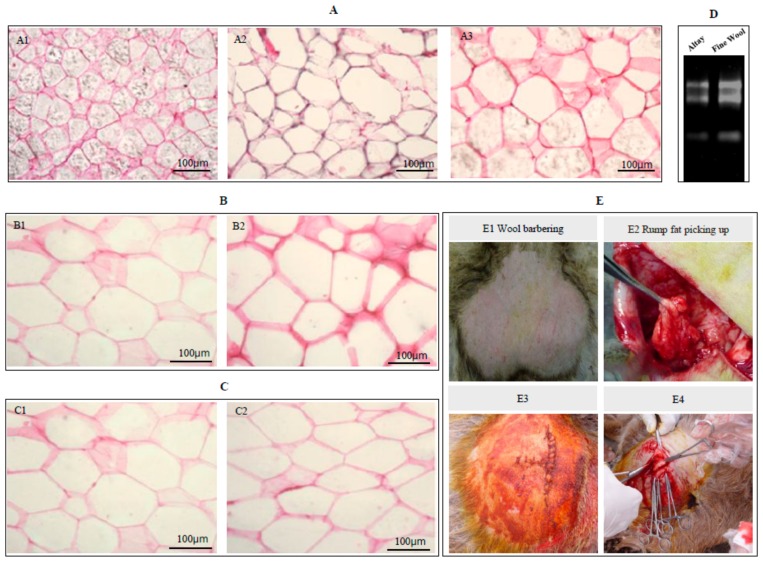
Frozen sections of sheep adipose tissue and fat removal procedure. (**A**) Frozen sections of Altay sheep adipocytes at distinct stages of the life cycle. (**A1**) Young Altay sheep, (**A2**) Six-month-old Altay sheep, (**A3**) Adult Altay sheep. (**B**) Frozen sections of adult Altay and Wool sheep. (**B1**) Adult Altay sheep, (**B2**) Adult Wool sheep. (**C**) Frozen sections of tail adipose tissue from free-feeding and starvation-subjected Altay sheep. (**C1**) Free-feeding group, (**C2**) Persistent starvation group. Sections were observed under a microscope with a 40× objective. (**D**) Equivalent adipose tissue amounts from Altay and Wool sheep for extraction of RNA. Equivalent masses of adipose tissue from Altay sheep (28S and 18S RNA) were darker than those of Wool sheep, suggesting that the adipocytes of Altay sheep contain larger amounts of lipid droplets and less cytoplasm. (**E**) Fat removal surgery in Altay sheep. After barbering the wool, the rump fat was removed, and the skin sutured. After several months, the rump was restored, suggesting that fat deposition is also dependent on the adipocyte number.

The adipose tissue from all groups were collected via operative procedures, and the relative frozen sections were examined. The examination of the size of the adipocytes from the tail fat of Altay sheep in each age group (young lamb, six months old, and adult) revealed an increase in adipocyte volume with age (Figure 2A). Within the same unit area, the number of adipocytes in young lamb, six-month-old sheep, and adult sheep groups was determined as 56, 33, and 26, respectively, with the cell volumes of six-month-old and adult sheep being significantly larger than those of young lambs. We then compared the adipocyte size of Altay sheep with that of Wool sheep. The results showed that the adipocytes of Altay sheep tail fat displayed a nearly circular cell morphology with relatively uniform sizes, in contrast to those of the control group (Wool sheep) that showed a non-uniform, irregular morphology. Within the same unit area, the number of adipocytes in Altay and Wool sheep was estimated as 14 and 18, respectively. The cell-filling degree was markedly higher in Altay than in Wool sheep (Figure 2B). Meanwhile, six selected healthy Altay sheep of similar weights were subdivided into FF and PS groups. The adipose tissue from both groups was collected via operative procedures, and the related frozen sections were examined. Within the same unit area, the number of adipocytes in the FF and PS groups was estimated as 14 and 20, respectively. Moreover, the cells of sheep from the PS group were flat and spindle-shaped, with a lower filling degree than those from the FF group (Figure 2C), suggesting a significant decomposition of intracellular lipids. On the basis of these data, we propose that the persistent starvation model developed in this study achieved the expected effect and effectively simulated the deposition and mobilization of tail fat in Altay sheep in the natural environment.

### 2.4. iTRAQ Analysis of Proteins Participating in Altay Sheep Rump Fat Metabolism under Persistent Starvation

**Figure 3:**
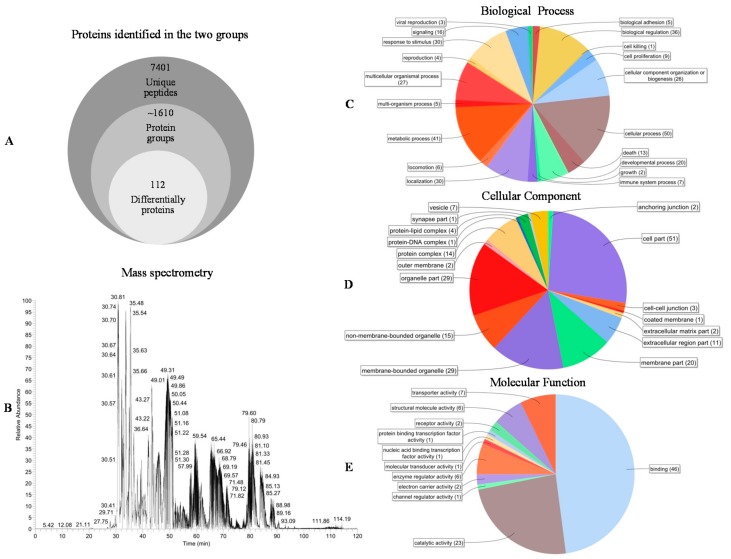
Protein classification according to molecular function. (**A**) In total, 7401 unique peptides, about 1610 protein, and 112 differentially expressed proteins were identified in the tail adipose tissue of Altay sheep from the FF and PS groups using iTRAQ combined with LC–MS/MS technology. (**B**) Total ion flow map of mass spectrometry. (**C**) Biological process-based classification of the about 1610 proteins. (**D**) Cellular component-based classification of the about 1610 proteins. (**E**) Molecular function-based classification of the about 1610 proteins.

To profile global protein expression changes in the rump in response to tail fat mobilization, we subjected Altay sheep to persistent starvation (Figure S1A). The proteomes of Altay sheep rump adipose tissues from the FF and PS groups were compared using three independent samples as biological replicates. After digestion of the extracted proteins with trypsin, purified Tandem Mass Tag-labeled peptides from the FF and PS replicates were pooled. All samples were analyzed via LC–MS/MS, and the raw spectra were processed using MaxQuant (Figure S1B). Consequently, we identified 7401 unique peptides and about 1610 proteins in rump adipose tissues labeled with each channel with high confidence, which were subsequently classified into 14 functional categories according to gene ontology (Figure 3). Among the proteins detected, 112 were differentially expressed (Table 1). Compared with the FF group, 54 proteins were downregulated and 58 were upregulated in the PS group. For instance, the expression of ADIPOQ and complement factor D (CFD) was higher in the PS than in the FF group, while Cell death-inducing DFFA-like effector C (CIDEC) expression in the PS group was downregulated to a greater extent than in the FF group. A number of genes closely related to lipid metabolism were further screened (Table 2). Additionally, we performed BLAST analysis of target protein sequences using the KEGG genes database of goat protein sequences, followed by grouping of the 112 identified protein sequences into 99 KEGG pathways for pathway analysis and screening of individual pathways related to fat deposition and mobilization (Table 3).

### 2.5. qPCR Analysis of Differentially Expressed Candidate Genes

**Figure 4:**
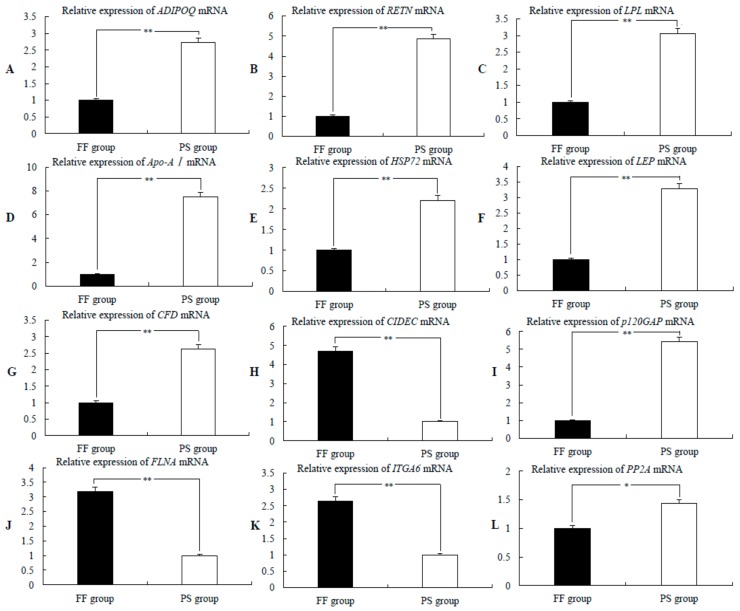
Expression and analysis of candidate genes related to fat development. (**A**–**L**) qPCR analysis of the expression patterns of lipid metabolism-related candidate genes adiponectin (*ADIPOQ*), resistin (*RETN*), lipoprotein lipase (*LPL*), apolipoprotein AI (*Apo-AI*), heat-shock protein 72 (*HSP72*), leptin (*LEP*), complement factor D (*CFD*), and cell death-inducing DFFA-like effector C (*CIDEC*)) and non-lipid metabolism-related candidate genes p120 GTPase activating protein (*p120GAP*), filamin A (*FLNA*), integrin alpha 6 (*ITGA6*), and protein phosphatase 2A (*PP2A*)) in the free-feeding (FF) and persistent starvation (PS) groups (* *p* < 0.05, ** *p* < 0.01).

To explore the expression patterns of fat synthesis and decomposition-related candidate genes, we exposed sheep to persistent starvation and free-feeding conditions to simulate the two extreme states promoting tail fat mobilization and deposition. qPCR was applied to analyze the expression of fat synthesis- and decomposition-related candidate genes in Altay tail fat before (FF group) and after (PS group) the starvation test, with glyceraldehyde 3-phosphate dehydrogenase (*GAPDH*) as the reference gene. The relative expression levels of fat metabolism genes were calculated using the 2^−ΔΔ*C*T^ method. The levels of *ADIPOQ* (Figure 4A), *RETN* (Figure 4B), *LPL* (Figure 4C), *Apo-AI* (Figure 4D), *HSP72* (Figure 4E), *LEP* (Figure 4F), and *CFD* (Figure 4G) in the PS group were significantly higher than those in the FF group (*p* ≤ 0.01), while *CIDEC* (Figure 4H) expression was markedly lower in the PS group (*p* ≤ 0.01). Simultaneously, we examined the expression patterns of non-lipid metabolism-related genes. The expression of p120 GTPase activating protein (*p120GAP*) (Figure 4I) in the PS group was significantly higher than that in the FF group (*p* ≤ 0.01), while filamin A (*FLNA*) (Figure 4J) and integrin alpha 6 (*ITGA6*) (Figure 4K) levels were significantly higher in the FF group (*p* ≤ 0.01), and protein phosphatase 2A (*PP2A*) (Figure 4L) level was relatively higher in the FF group (0.01 ≤ *p* ≤ 0.05) than in the PS group. The qPCR results disclosed consistent mRNA and protein expression patterns of these genes, supporting the accuracy of our iTRAQ-derived proteomics data. Our findings indicate that both transcriptional and proteomic groups are involved in the adipose biology processes of tail fat deposition and mobilization in the two extreme states.

## 3. Discussion

### 3.1. Altay Sheep Present an Ideal Model for Studying Animal Fat Metabolism

Altay sheep have evolved into a fat-rump breed with superior environmental adaptability, naturally selected by lengthy environmental stress and human factors. Owing to their characteristic rump fat deposition and mobilization in response to seasonal changes, Altay sheep present an ideal model to investigate the molecular mechanisms underlying subcutaneous fat deposition and mobilization processes in the tails of animals. In this study, we recorded rump widths (cm) of Altay sheep from three age groups (three months old, yearling, adult). Rump width decrease was not obvious in three-month-old sheep, which may be attributable to their strong growth tendency. We additionally monitored blood lipid markers for one year in three-month-old, yearling, and adult Altay sheep. The TG, TCH, and FFA levels in Altay sheep from all three stages were relatively constant, with no significant differences among the groups (*p* ≥ 0.05). Our results indicate that the blood lipid markers fluctuate within the normal range during continuous fat deposition in healthy sheep. The sectioning and hematoxylin-eosin (HE) staining results showed that, with increasing age, rump adipocyte volume and width are correspondingly increased. Cell size was positively correlated with the fat deposition ability, in keeping with the earlier finding that adipocyte volume increases with age in animals [20]. In addition, the volume of tail adipocytes of Altay sheep was significantly larger than that of low-fat wool sheep at the same growth stages. These data clearly show that Altay sheep have a stronger ability to deposit tail fat than wool sheep.

To further clarify the specific mechanisms underlying fat mobilization, Altay sheep were subjected to persistent starvation involving a progressive simulation of fat mobilization through food restriction. In the PS group, the degree of filling of the tail adipocytes was significantly lower than that in the FF group, consistent with the findings of Gondret et al. [21]. These results support the theory that in Altay sheep, rump decomposition is accelerated, and storage fat consumed to produce heat and energy that promote resistance to cold weather and facilitate the maintenance of body temperature and physiological activities under conditions of persistent starvation. 

### 3.2. Roles of Specific Hormones in Lipid Metabolism

We identified 7401 unique peptides and about 1610 proteins in the FF and PS groups using iTRAQ protein sequencing. Overall, 112 differentially expressed proteins were identified in the two groups. Among these, the known fat-specific hormones LEP and ADIPOQ displayed increased secretion in the persistent starvation state (*p* ≤ 0.01). These two proteins are able to suppress the appetite of Altay sheep by inhibiting the secretion of hypothalamus neuropeptide Y and reducing energy intake and to increase the expression of lipolytic enzymes via the AMPK pathway to promote fat mobilization [22,23]. LEP is reported to stimulate the central glucosensory neurons, increase sympathetic nerve activity, and promote the release of peripheral noradrenaline to enhance the secretion of lipolysis hormones and accelerate lipid droplet decomposition. In addition, ADIPOQ activates the p38-MAPK and PPARα signal pathways to increase energy metabolism [24]. The combined action of these two hormones may ultimately accelerate the decomposition of rump fat and promote the oxidation of fatty acids to supply energy.

### 3.3. Synergistic Actions of Key Lipid Metabolism-Related Hormones and Signaling Pathways 

Under conditions of persistent starvation, the activation of specific signaling pathways closely related to lipid metabolism constitutes another main mechanism for promoting rump decomposition to supply energy in Altay sheep. *Apo-AI* and *LPL* mRNA and protein levels in the PS group were significantly higher than in the FF group (*p* ≤ 0.01). These two differentially expressed proteins participate in the PPAR pathway and have been identified as downstream target genes of PPARα and PPARβ, respectively. *LPL* is reported to promote the exchange between lipoproteins and lipids through the regulation of triglyceride hydrolysis and assist in lipoprotein intake and the acceleration of fatty acid oxidation rate [25,26]. Apo-AI is the main component of plasma high-density lipoproteins that accelerate the cholesterol transfer rate and the decomposition rate of lipids [27]. The two proteins promote fatty acid β-oxidation. The differentially expressed LEP and ADIPOQ proteins in our experiments were simultaneously enriched in the AMPK signaling pathway. Both proteins increase energy metabolism by controlling fatty acid oxidation, glucose intake, and lipolysis by activating the AMPK signal pathway [28]. The resulting activation of these signaling mechanisms and the release of lipolysis-related hormones trigger a positive feedback pathway that enhances the activities of downstream genes regulating energy metabolism, further promotes catabolism and lipid mobilization, and accelerates the oxidation of fatty acids to supply energy and maintain the homeostasis of the energy metabolism in sheep under the persistent starvation state. 

### 3.4. Inhibition of Lipid Droplet Fusion Ability in Combination with Enhanced Lipolysis

Both promotion of lipolysis and inhibition of fat deposition could lead to the reduction of rump fat accumulation in Altay sheep. The CIDEC protein is reported to promote fat accumulation and inhibit lipolysis, and knockout of the corresponding gene leads to a significant increase in the decomposition of TG in adipocytes and to accelerated energy metabolism [29,30,31]. In our experiments, *CIDEC* mRNA and protein levels were significantly lower in the PS group, than in the FF group (*p* ≤ 0.01). These results support the theory that the downregulation of CIDEC inhibits lipid droplet fusion and delays the rate of fat deposition by blocking triglyceride aggregation, consistent with the sectioning and HE staining data obtained from Altay sheep rump adipocytes. Accordingly, we propose that persistent starvation in a harsh environment eventually leads to the withering of Altay sheep rump through combined inhibition of lipid synthesis and acceleration of fat decomposition.

### 3.5. Activation of Critical Genes for Maintaining Internal Homeostasis

#### 3.5.1. Insulin Resistance Promotes Lipolysis

Insulin is reported to downregulate blood glucose to promote fat synthesis [32]. RETN and HSP72 are adipocyte factors associated with insulin resistance. The expression of these factors at both the mRNA and protein levels was significantly higher in the PS than in the FF group (*p* ≤ 0.01). Our results indicate that under the persistent starvation state, the expression of these proteins is correlated to increased fat mobilization, promotion of energy release, and decomposition of rump fat in Altay sheep. On the other hand, these proteins could enhance insulin resistance, facilitating the maintenance of a constant blood glucose level, and provide protection for complex cell types and organ systems, such as the nervous system, blood cells, and the immune system, thus preventing the disruption of their physiological functions. These findings further support the association of the *RETN* and *HSP72* genes with insulin resistance [33,34,35]. In addition, *CFD* expression in the PS group was significantly higher than in the FF group (*p* ≤ 0.01), in keeping with a previous report by Fan et al. [36] that *CFD* mRNA is augmented in the catabolic state. On the basis of the collective data, we speculate that upregulation of CFD promotes lipolysis via insulin resistance. However, further studies are required to elucidate the precise mechanisms.

#### 3.5.2. Initiation of Body Stress Mechanisms

Persistent starvation usually triggers a series of abnormal reactions in the animal body, such as decreased heat production, hypothermia, decreased immunity, neuroendocrine dysfunction, and body fat consumption [37]. Altay sheep subjected to persistent starvation showed consumption of body fat but no obvious immune disorders or neurological abnormalities, which may be attributable to the activation of specific genes involved in body self-protection and repair [38,39]. Our iTRAQ results revealed significantly higher expression of HSP72 and Apo-AI in the PS group, compared to the FF group (*p* ≤ 0.01). The related genes are reported to exert anti-inflammatory and antioxidant effects [40,41]. We hypothesize that, as a result of persistent starvation, HSP72 and Apo-AI are upregulated, activating the body stress mechanisms, and act to effectively reduce oxidative stress. These proteins contribute to cell protection and reduce inflammation, enhance body resistance, and reduce the likelihood of disease caused by malnutrition. Consequently, Altay sheep adapt to a series of adverse reactions caused by persistent starvation and are able to maintain internal homeostasis under harsh environmental conditions.

**Figure 5:**
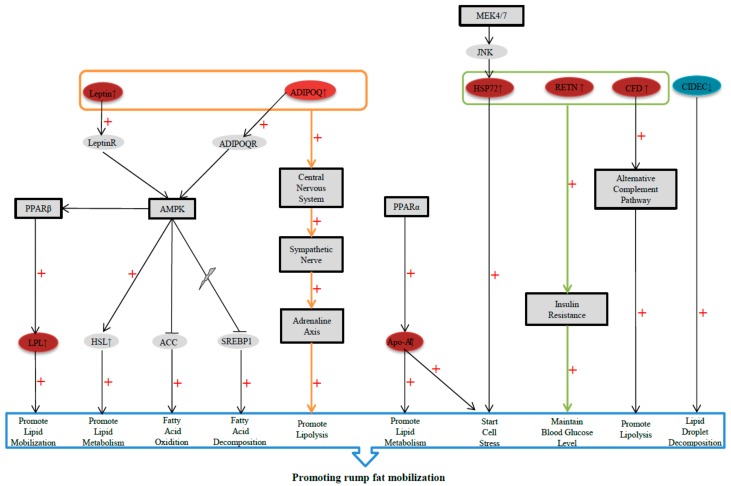
Genes and signaling pathways involved in rump fat mobilization in response to continuous starvation. Under persistent starvation conditions, the synergistic actions of lipid metabolism-related hormones (LEP and ADIPOQ), key signaling pathways of fat mobilization and fatty acid oxidation (AMPK and PPAR), the fat synthesis-related gene *CIDEC*, insulin resistance-related genes (*HSP72*, *RETN*, and *CFD*), and antioxidant and anti-inflammatory genes (*HSP72* and *Apo-AI*) provide a molecular basis and material guarantee for improving the adaptability of Altay sheep to extreme environments. Red represents upregulated genes and blue represents downregulated genes; “+” promotion.

In summary, the extreme environment characterized by persistent cold and starvation stimulates the central nervous system in Altay sheep, leading to increased secretion of serum LEP and ADIPOQ. These hormones act on adipocytes through the AMPK signaling pathway and sympathetic adrenal axis to promote tail fat decomposition. Simultaneously, the expression levels of specific genes related to lipid metabolism are upregulated, such as *Apo-AI*, *LPL*, *LEP*, and *ADIPOQ*. These genes activate the AMPK and PPAR signaling pathways and form a positive loop to accelerate adipocyte decomposition. In addition, the *CIDEC* gene is downregulated, resulting in the inhibition of lipid droplet fusion and reduced accumulation of tail fat. We simultaneously identified a number of genes that play important roles in fat mobilization and internal homeostasis. (a) *RETN*, *HSP72*, and *CFD* were upregulated, which could enhance insulin resistance, accelerate tail fat decomposition and fatty acid oxidation, as well as effectively maintain a normal blood glucose level to provide protection to complex cell types and organ systems, such as the nervous system, blood cells, and the immune system, and enable their normal physiological functions under stress conditions. (b) *HSP72* and *Apo-AI* were upregulated, leading to the activation of stress mechanisms, the promotion of cell protection, and the reduction of inflammation, and consequently improving the adaptability of Altay sheep to persistent starvation. Thus, under environmental stress conditions, such as cold and starvation, the secretion of lipid metabolism-related hormones is increased, the key signaling pathways of fat mobilization and fatty acid oxidation are activated, and the effects of insulin resistance are enhanced, along with the simultaneous inhibition of fat synthesis, to ultimately accelerate Altay sheep tail fat decomposition and supply energy. During this process, a constant blood glucose level and stress state in the cells is maintained, and antioxidant and anti-inflammatory activities are enhanced (Figure 5). These regulatory processes collectively provide a molecular basis and material guarantee for improving the adaptability of Altay sheep to extreme environments. Our experiments additionally revealed significant changes in the expression of non-lipid metabolism-related genes, such as *p120GAP*, *PP2A*, *FLNA*, and *ITGA6*. The mRNA levels of each gene were consistent with the protein expression patterns. However, further research is warranted to ascertain whether these genes are involved in maintaining internal homeostasis in extreme states in Altay sheep as well as to understand their functions and molecular signaling.

## 4. Materials and Methods

### 4.1. Ethics Statement

Procedures were performed according to the Guide for the Care and Use of Laboratory Animals and approved by the Experimental Animal Care and Use Committee of Xinjiang Academy of Agricultural and Reclamation Sciences (Shihezi, China, ethic committee approval number: XJNKKXY-AEP-039, 22 January 2012).

### 4.2. Experimental Design

Three adult female Altay sheep in good physical condition with similar ages, body weights, and hip types were used. All animals were provided by the farm of Xinjiang Academy of Agricultural and Reclamation Sciences. All three sheep were given ample forage (500 g alfalfa and 200 g cottonseed hull) three times a day, with corn (200 g) as complementary feed at night, and water was provided ad libitum. After continuous feeding for four weeks, three adipose tissue samples from the left hip of each sheep were collected and represented the control group, designated FF. The wounds were sutured after sample collection, and the sheep were treated with antibiotics and provided food and drinking water ad libitum for a further four weeks. After their physiological conditions returned to the normal state, unit feed restriction was initiated (weekly feeding volume reduced by half along with ad libitum drinking water) for two weeks, followed by four weeks of complete fasting with unlimited drinking water. At the end of the starvation period, adipose tissue samples from the right hip were collected, representing the test group, designated PS.

The rump adipose tissue samples from each subgroup (FF and PS) were snap-frozen in liquid nitrogen and stored at −80 °C until use in iTRAQ proteomic and qPCR analyses for the identification and validation of protein factors and candidate genes.

### 4.3. Altay Sheep Rump Width Distribution

Rump widths of nine Altay sheep from three age groups (three months old, yearling, adult) were recorded monthly from May 2012 to May 2013.

### 4.4. Determination of the Serum Lipid Markers in Altay Sheep

The sera of nine Altay sheep from the three age groups (three months old, yearling, adult) were collected at 10 am on day 9 of every month (between August 2012 and July 2013) and stored at −20 °C for the evaluation of serum lipid markers (APN, LEP, TG, TCH, and FFA) using enzyme-linked immunosorbent assay (ELISA, Blue Gene, Shanghai, China). The absorption values at 450 nm were assessed using an Enzyme Mark Instrument (Thermo Scientific, Waltham, MA, USA). 

### 4.5. Frozen Sections and Hematoxylin-Eosin Staining

Tail fat tissue samples of nine Altay sheep from three age groups (young lamb, six months old, adult), three Wool sheep (adult), and six Altay sheep from the FF and PS groups were used to generate frozen sections 6 µm thick. One ethanol-fixed tail fat tissue sample from each subgroup was randomly selected for HE staining, sealed with neutral balata, and observed under a microscope.

### 4.6. iTRAQ Sample Preparation

Six frozen tail fat tissue samples from the two subgroups (FF and PS) were transferred into SDT buffer (4% sodium dodecyl sulfate (SDS), 1 mM dithiothreitol (DTT), 150 mM Tris HCl, pH 8.0) and sonicated (80 W, ultrasonic 10 s, intermittent 15 s, a total of 10 times) on ice. After centrifugation at 14,000× *g* for 10 min, protein concentration was determined from an aliquot of supernatant with the Bicinchoninic acid assay. Each protein sample (60 μg) was digested with a trypsin solution (50 μg/mL) at 37 °C for 16 h. Following trypsin digestion, the peptides were dried via vacuum centrifugation and processed with the 8-plex iTRAQ reagent (Applied Biosystems, Foster City, CA, USA) as follows: FF 1 (113 tag), FF 2 (114 tag), FF 3 (114 tag), PS 1 (115 tag), PS 2 (116 tag), PS 3 (118 tag), and sample mix (119 tag), according to the manufacturer’s instructions. After tagging, all samples were pooled, purified using Waters SepPak tC18 cartridges (Waters, Milford, MA, USA), and subjected to SCX fractionation with buffer A (10 mM KH2PO4, pH 3.0, 25% ACN) and buffer B (10 mM KH2PO4, pH 3.0, 500 mM KCl, 25% ACN). Tagging efficiency of >99% was confirmed via mass spectrometry.

### 4.7. LC and MS/MS Analyses

The iTRAQ-labeled peptide mixtures were separated using an Easy LC nano-HPLC liquid-phase system with tray cooling comprising Buffer A (0.1% formic acid (FA)) and Buffer B (84% acetonitrile (ACN) and 0.1% FA). The peptides were loaded on a 2 cm × 100 μM and 75 μM × 100 mm EASY column (Thermo Scientific, Waltham, MA, USA), both filled with a 3 μM C18 trap column. The peptides were eluted in a linear gradient with buffer B from 0% to 35% over 100 min at a flow rate of 250 nL/min. MS analysis was performed on the liquid chromatographic eluent at a mass spectral range of 300–1800 m/*z* and accumulation time of 60 ms per spectrum.

### 4.8. Database Searches and Analysis of the Identified Proteins

Analyst Mascot 2.2 and Proteome Discoverer 1.4 (Thermo Scientific, Waltham, MA, USA) software were used to acquire the iTRAQ-based proteomic data. Paragon database search algorithm and integrated false discovery rate (FDR) analysis were implemented in ProteinPilot software (Applied Biosystems, Foster City, CA, USA) for peptide identification. A 99% confidence interval (CI) was set as the significance threshold for protein identification. The confidence level of each differentially expressed protein was calculated as a p-value using ProteinPilot, allowing the results to be evaluated on the basis not only of the magnitude but also of the confidence level of the change. Gene ontology (GO) was performed using the bioinformatics analysis tool DAVID (http://david.abcc.ncifcrf.gov) for the functional classification of the iTRAQ-identified proteins.

### 4.9. qPCR Analysis

qPCR was performed to validate the iTRAQ-based results. The expression levels of candidate genes in the FF and PS groups of Altay sheep were confirmed using the qPCR assay based on SYBR Green fluorescence (primer sequences are presented in Table 4). The experiments were repeated three times with double-distilled water set as the negative control.

### 4.10. Data Analysis

One-way ANOVA was conducted to evaluate changes between the FF and PS groups. Dependent variables were examined in terms of normality of variance. The effects of single treatments were analyzed with post-hoc tests at the same probability level after ANOVA was performed. For qPCR data analysis, the 2^−ΔΔ*C*t^ method was used to calculate the relative expression of candidate genes.

## Figures and Tables

**Table 1:** Identification data related to the 112 proteins differentially expressed in the normally fed and starved groups.

Accession No.	Gene	Protein Description	Coverage	Peptides	Theoretial MW/pI	FF/PS	*p*-Value
Q5E9M1	*ARL6IP5*	PRA1 family protein 3	18.09	3	21.6/9.49	1.280389	0.001434
F1MMU4	*H1FX*	H1 Histone Family, Member X	7.01	1	22.3/10.71	2.019082	0.003628
G1DFT2	*RPS4X*	Ribosomal Protein S4, X-Linked	9.89	3	29.6/10.25	1.279684	0.0069
Q3T0S6	*RPL8*	60S ribosomal protein L8	16.34	4	28.0/11.03	1.292493	0.007701
D7R7V6	*GAPDH*	Glyceraldehyde-3-phosphate dehydrogenase	42.94	14	35.8/8.35	1.174065	0.007889
W5PGZ8	*APOF*	Apolipoprotein F	4.32	1	35.4/8.06	1.546838	0.00792
W5PHJ8	*RP*	40S ribosomal protein	13.42	3	26.0/9/96	1.149951	0.008232
A5D9B4	*HNRPH2*	Heterogeneous nuclear ribonucleoprotein H2	7.57	3	49.2/6.30	1.158449	0.008327
F1MN90	*CIDEC*	Cell death-inducing DFFA like effector c	6.84	1	27.3/7.97	1.224292	0.008439
L8IUX9	*M91_16660*	Cytochrome b-c1 complex subunit 8	8.54	1	9.6/10.33	1.228296	0.009478
F1MXT1	*HACD2*	Very-long-chain (3*R*)-3-hydroxyacyl-CoA dehydratase	8.27	2	28.4/9.56	1.331041	0.010441
W5QBL2	*CUL1*	Cullin 1	1.05	1	87.4/8.22	1.225131	0.012333
P20072	*ANXA7*	Annexin A7	6.7	3	49.9/6.80	1.252655	0.0138
Q9XSK2	*CD63*	CD63 Molecule	4.2	1	25.7/7.64	1.750327	0.01455
W5PED5	*CYP4V2*	CytochromeP450	7.78	3	61.0/7.15	1.417145	0.016098
A4ZYA6	*VIM*	Vimentin	72.75	37	53.6/5.09	1.370539	0.017049
W5NY07	*ATL3*	Atlastin GTPase 3	11.5	6	60.0/5.71	1.437302	0.018839
Q8WN55	*PTBP1*	Polypyrimidine tract-binding protein 1	8.85	4	57.0/9.17	1.142094	0.021158
L8I566	*M91_19776*	Coatomer subunit alpha	1.07	1	137.7/7.29	1.330717	0.021314
F4YD28	*RPS24*	40S ribosomal protein S24	7..23	1	9.5/10.69	1.159825	0.022228
W5P7F8	*TSPAN*	Tetraspanin	4.24	1	25.8/8.10	1.713756	0.022317
W5PK26	*LASP1*	LIM and SH3 Protein 1	10	2	29.6/7.04	1.619281	0.02251
A8D8X1	*RPL10*	Ribosomal Protein L10	18.69	5	24.5/10.14	1.363970	0.0233
D3YC70	*HB*	Heparin-binding protein	25	1	5.6/8.61	1.230956	0.024758
C3V9V7	*RNP24*	Ribonuclear protein 24	7.46	2	22.7/5.17	1.427028	0.024858
W5PSJ3	*DCTN1*	Dynactin 1	2.62	3	143.0/5.85	1.184554	0.025664
F1MBS3	*TGFBI*	Transforming growth factor-beta- induced protein ig-h3	13.16	7	72.2/7.12	1.500014	0.026213
W5NSV5	*ITGA6*	Integrin Alpha 6	4.28	4	106.5/7.43	1.246541	0.026711
E3SAZ8	*NPM1*	Nucleophosmin	12.07	4	32.1/4.85	1.283429	0.02941
Q307E6	*COL1A1*	Collagen, Type 1, Alpha 1	8	1	15.5/9.12	3.591183	0.0297
L8IR22	*M91_01904*	Basigin	16.61	4	29.7/5.93	1.164516	0.0299
Q5E971	*TMED10*	Transmembrane emp24 domain- containing protein 10	4.11	1	24.8/6.68	1.485596	0.03096
W5PIP4	*AZU1*	Azurocidin 1	4.45	1	26.6/11.13	1.673528	0.031409
Q30B83	*RPL35a*	Ribosomal protein L35a	14	1	5.8/9.78	1.211594	0.031542
L8IPP0	*M91_20624*	78 kDa glucose-regulated protein	36.03	19	72.3/5.16	1.105556	0.031702
W5NSY8	*SH3BGRL*	SH3 domain-binding glutamic acid-rich- like protein	64.65	4	11.2/5.04	1.147976	0.031807
C5IJ89	*RHEB*	Ras Homolog Enriched In Brain	7.61	2	20.4/5.92	1.151032	0.0318
W5QF34	*LMF2*	Lipase maturation factor	2.15	1	72.8/9.96	1.289405	0.034567
D6BRG0	*ITGB*	Integrin, Beta	2.76	2	84.3/5.24	1.703519	0.0359
L8HQV0	*M91_13225*	Dehydrogenase/reductase SDR family member 1	8.01	2	34/8.36	1.316582	0.03605
A1L555	*PSAP*	Prosaposin	1.9	1	58.1/5.24	1.542171	0.03648
W5P5A0	*FLNA*	Filamin A, Alpha	10.82	23	279.6/6.27	1.077701	0.0336
L8IBL4	*M91_10571*	Lipoma-preferred partner	1.91	1	49.9/7.78	1.143675	0.037407
K0IVJ6	*metallothionein*	Metallothionein	26.09	1	4.3/8.23	1.870961	0.038453
W5PJD0	*PGM2*	Phosphoglucomutase 2	1.2	1	64.9/6.44	1.18212	0.039518
W5P263	*ABHD5*	alpha/beta hydrolase domain-containing protein 5	2.4		37.2/8.10	1.399659	0.040579
L8HSP5	*M91_02935*	Basic leucine zipper and W2 domain-containing protein 1	6.86	3	48.4/5.77	1.529994	0.042248
P07514	*CYB5R3*	NADH-cytochrome b5 reductase 3	44.19	11	34.0/7.28	1.304232	0.042261
W5NVF1	*CYBRD1*	Cytochrome B Reductase 1	5.88	1	22.3/9.27	2.219898	0.042413
A6QQR5	*TMEM43*	Transmembrane Protein 43	9	3	44.7/8.70	1.578646	0.044298
W5QHC9	*EPB42*	Erythrocytemembrane protein band 42	11.87	8	77.2/6.85	1.263628	0.04501
Q3SZF2	*ARF4*	ADP-Ribosylation Factor 4	35	2	20.5/6.18	1.683021	0.0453
L8IHC9	*M91_15717*	Uncharacterized protein	11.11	1	6.2/9.48	1.095345	0.045529
W5PN85	*ACAT1*	Acyl-coenzyme A:cholesterol acyltrans- ferase	24.36	9	45.4/8.55	1.382609	0.049793
W5Q2N1	*p120GAP*	Ras GTPase-activating protein	1.19	1	106.0/7.76	0.733429	0.00144
A0FI82	*LPL*	Lipoprotein lipase	5.65	2	53.5/8.36	0.701299	0.004433
W5QA54	*LOC101119629*	Uncharacterized protein	7.08	1	22.4/5.31	0.273731	0.005858
P02510	*CRYAB*	Alpha-crystallin B chain	25.14	4	20.0/7.32	0.749447	0.006911
B0BL70	*mbl*	Mannose-binding lectin	4.42	1	26.5/4.89	0.626459	0.008106
Q1JPJ2	*XPNPEP1*	Xaa-Pro aminopeptidase 1	3.53	2	69.7/5.68	0.860442	0.008278
Q2KIL3	*ALAD*	Delta-aminolevulinic acid dehydratase	10.03	2	36.1/6.99	0.570859	0.008597
F1N2I5	*CMBL*	Carboxymethylenebutenolidase	8.16	2	27.9/7.07	0.675716	0.009004
L8IMU6	*PP2A*	Protein Phosphatase 2 Alpha	8.3	1	28.7/5.43	0.719336	0.009013
L8IEF9	*M91_08893*	Vacuolar protein sorting-associated protein 28 homolog	5.94	1	22.7/8.42	0.656248	0.009294
A7XV32	*HSP72*	Heat Shock Protein 72	28.24	18	70.3/5.65	0.806279	0.010322
A0A0U1YZ59	*ADIPOQ*	Adiponectin	31.8	5	25.9/6.3	0.764493	0.0107
H9A6H7	*MSTN*	Myostatin	6.17	1	9.3/5.17	0.294348	0.0127
W5PCD1	*LOC101110151*	Uncharacterized protein	19.15	3	15.3/5.19	0.771917	0.013342
F1N5Q0	*ST*	Sulfotransferase	3.14	1	26.5/8.83	0.793002	0.013347
W5QGT1	*CUL3*	Uncharacterized protein	2.68	2	86.4/7.98	0.79521	0.013851
B6UV62	*SERPINF1*	SERPINF1	18.03	7	45.9/7.95	0.624886	0.014795
W5NX51	*APOA1*	Apolipoprotein A-I	65.64	22	29.5/6.20	0.489784	0.015501
L8IVL6	*M91_02549*	Glutamate-cysteine ligase catalytic subunit	6.3	3	66.6/5.57	0.65182	0.015547
L8IZ67	*M91_00380*	Glutaredoxin-3	4.19	1	35.0/6.18	0.715634	0.015974
Q762I5	*RETN*	Resistin	18.74	1	5.6/8.69	0.799622	0.0167
W5QDD0	*LOC101108092*	Uncharacterized protein	59.83	15	26.7/7.00	0.737769	0.017758
Q3T0A3	*CFD*	Complement factor D	67.85	2	61.1/7.53	0.698614	0.018044
P29701	*AHSG*	Alpha-2-HS-glycoprotein	46.15	12	38.6/5.37	0.720115	0.01812
W5P8E1	*DNPH1*	2′-deoxynucleoside 5′-phosphate *N*-hydrolase 1	5.75	1	18.7/5.40	0.833179	0.020246
Q32LE5	*ASRGL1*	Isoaspartyl peptidase/l-asparaginase	6.82	2	32.0/7.40	0.730442	0.020663
D6PZY4	*fH*	Factor H	9.09	10	125.2/6.71	0.772665	0.020943
W5PJ97	*APOA2*	Apolipoprotein A-II	25.49	2	11.2/8.10	0.456384	0.02237
W5Q961	*LOC101107947*	Uncharacterized protein	7.48	2	28.3/5.79	0.67714	0.022512
L8IFD5	*M91_10356*	Guanine nucleotide-binding protein subunit alpha-13	5	2	44.3/7.22	0.837336	0.024252
W5Q0Y4	*FAH*	Fumarylacetoacetase	12.25	5	43.9/7.40	0.937515	0.024511
W5P214	*TAGLN*	Transgelin	40.89	8	25.0/8.95	0.701171	0.02459
I1U3B9	*ALDH1L1*	Aldehyde Dehydrogenase 1 Family, Member L1	4.08	1	56.1/7.63	0.670099	0.0248
Q3SZA0	*COPS4*	COP9 signalosome complex subunit 4	11.58	4	46.2/5.83	0.838176	0.024938
L8I8L3	*M91_03748*	Vesicle-associated membrane protein 4	5.04	1	16.1/6.04	0.475656	0.026818
A1L5B0	*MYL9*	Myosin regulatory light polypeptide 9	39.88	5	19.3/4.85	0.825417	0.028267
W5Q7J0	*APOB*	Apolipoprotein B	7.65	30	513.8/7.12	0.779207	0.02886
L8I9W2	*M91_02159*	Tyrosine-protein phosphatase non- receptor type 11	3.54	2	67.8/7.16	0.931432	0.032163
I1W1N3	*RAB18*	Member RAS Oncogene Family	5.34	1	22.9/5.23	0.815468	0.0336
W5PE22	*GDI2*	Rab GDP dissociation inhibitor	35.58	13	48.8/6.97	0.841934	0.034471
Q28603	*LEP*	Leptin	3.79	4	16.1/5.71	0.792210	0.0352
W5Q8X9	*PGP*	P-glycoprotein	5.91	1	25.1/5.28	0.776609	0.035498
W5NTW3	*ITIH1*	Inter-alpha-trypsin inhibitor heavy chain H1	12.06	9	101.5/7.26	0.530584	0.037196
C6ZP47	*HBA1*	I alpha globin	95.77	1	15.1/8.67	0.508000	0.0377
W5PFC9	*LOC101117129*	Uncharacterized protein	28.09	15	77.8/6.60	0.670015	0.039088
W5NZJ1	*LOC101114075*	Sulfotransferase	12.54	4	34.3/6.94	0.69946	0.039392
W5PR48	*HPRT1*	Hypoxanthine-guanine phosphoribosyltransferase 1	13.76	3	24.6/7.09	0.708477	0.039453
A6H7G2	*DBNL*	Drebrin-like protein	4.73	1	47.69/4.98	0.635779	0.041767
L8I9D6	*M91_05702*	B-cell lymphoma 3 protein	3.66	1	40.1/7.18	0.428236	0.041814
W5NR71	*DIAPH1*	Diaphanous 1	1.36	1	138.4/5.51	0.764331	0.042049
B9VH04	*UQCRH*	Cytochrome b-c1 complex subunit 6	28.57	2	10.6/4.50	0.811089	0.042158
P15497	*APOA1*	Apolipoprotein A-I	59.25	21	30.2/5.97	0.385227	0.04279
Q5E9A6	*VPS25*	Vacuolar protein-sorting-associated protein 25	8.52	1	20.7/6.34	0.794093	0.044223
W5PU66	*IQGAP1*	IQ motif containing GTP ase activating protein 1	8.87	12	192.8/6.37	0.908765	0.044485
L8IBI3	*M91_18442*	Terminal uridylyltransferase 7	1.08	1	168.8/7.06	0.390906	0.044636
W5P6Y6	*AK8*	Adenylatekinase 8	5.85	1	54.7/6.72	0.755031	0.046026
L8I977	*M91_12085*	Actin-related protein 10	3.48	1	44.7/7.62	0.791158	0.048268
W5QIC3	*PRUNE*	Uncharacterized protein	4.74	1	48.8/5.28	0.512771	0.048663

**Table 2:** Genes related to lipid metabolism.

Gene Name	Gene Ontology, Molecular Function
*GPIHBP1*	Lipase binding; Lipid binding; Lipoprotein particle binding;
*AHSG*	Glucose, Energy Metabolism; Lipid binding
*CYP450*	Cholesterol, steroids and other lipids synthesis
*CYB5R3*	Cholesterol biosynthetic
*HACD2*	Fatty Acyl-CoA Biosynthesis
*LPL*	Lipoprotein lipase activity; Triglyceride lipase activity; Phospholipase activity
*Apo-AI*	Lipid transporter activity; Phospholipid binding; Phospholipid transporter activity

**Table 3:** Proteins involved in the representative KEGG pathways.

Pathways	Proteins
PPAR signaling pathway	Apo-AI, LPL
AMPK signaling pathway	PP2A
PI3K–Akt signaling pathway	Cdc37, PP2A, ITGB, ITGA
Oxidative phosphorylation	UQCRH
MAPK signaling pathway	FLNA, HSP72, p120GAP
Glycerolipid metabolism	3.1.1.34

**Table 4:** Primer sequences and PCR product sizes.

Gene	Accession No.	Primer Sequence (5′→3′)	Product Size (bp)
*GAPDH*	NM_001190390	F: CTGACCTGCCGCCTGGAGAAA R: GTAGAAGAGTGAGTGTCGCTGTT	149
*ADIPOQ*	KM216385.1	F: AGTGGTGCCGTCATAGTGG R: CAGTGTAAATGGGGATGTGG	138
*RETN*	KJ704841.1	F: GCAGCACCTGCAGGATGAAG R: GTGGTCTCAGCACGCACGTC	263
*CFD*	XM015093908.1	F: TATCACGACGGCACCATCAC R: TACCGGGCTTCTTGCGGTTG	160
*FLNA*	FJ458435.1	F: CCATCGACGGGCCTTCCAAG R: TGGCAGTCTTGGTCAGGGAG	234
*p120GAP*	XM015096185.1	F: CATCCTGAGTCCACGGATGT R: GGAAGTTCAGGTACATTCCC	219
*PP2A*	XM012116023.2	F: GAGGGAATCATGAAAGCCGT R: CAGACCATAAGAGATCACAC	261
*LEP*	XM012177090.2	F: CCTATCTCTCCTACGTGGAGG R: GGATCTGTTGGTAGATTGCCA	213
*ITGA6*	XM012120035.2	F: GTGGCCATTCTTGCTGGGAT R: CTATCATCGTACCTAGAGCG	222
*LPL*	NM001009394.1	F: GCTGCTGGTATTGCAGGAAG R: CACTTCACTAGCTGGTCCAC	308
*HSP72*	NM001267874.1	F: ACCATCCCCACGAAGCAGAC R: CCTCGTCCTCTGCCTTGTAC	337
*Apo-AI*	XM012095497.1	F: GCCCAATTTGAAGCCTCCGC R: TCTGGCGGTAGATCTCCACC	246
*CIDEC*	KM199684	F: ATGGAATACGCCAAGAAGTC R: GGATTGGAAATACCCTTCTG	300

## Supplementary Materials

Supplementary materials are available online at http://www.mdpi.com/1422-0067/19/9/2556/s1.

## Abbreviations

iTRAQ	Isobaric peptide labeling approach
WAT	White adipose tissue
BAT	Brown adipose tissue
MSC	Mesenchymal stem cells
LC-MS	Liquid chromatography-mass spectrometry
AMPK	AMP-activated protein kinase
MAPK	Mitogen-activated protein kinase
ERK	Extracellular signal-regulated protein kinase
JNK	Extracellular signal-regulated protein kinase
p38 MAPK	p38 Mitogen-activated protein kinase
PPAR	Peroxisome proliferator-activated receptor
HPLC	High-performance liquid chromatography
GO	Gene ontology
KEGG	Kyoto Encyclopedia of Genes and Genomes
FDR	False discovery rate
ELISA	Enzyme-linked immunosorbent assay
HE	Hematoxylin-eosin
SDS	Sodium dodecyl sulfate
DTT	Dithiothreitol
FA	Formic acid
ACN	Acetonitrile
ANOVA	Analysis of variance
TG	Triglyceride
FFA	Free Fatty Acid
TCH	Total Cholesterol
LEP	Leptin
*GAPDH*	Glyceraldehyde 3-phosphate dehydrogenase
*Apo-AI*	Apolipoprotein AI
*ITGA6*	Integrin alpha 6
*CFD*	Complement factor D
*p120GAP*	p120 GTPase activating protein
*HSP72*	Heat-shock protein 72
*LPL*	Lipoprotein lipase
*PP2A*	Protein Phosphatase 2A
*ADIPOQ*	C1Q and collagen domain-containing, Adiponectin, APN
*CIEDC*	Cell death-inducing DFFA-like effector C
*FLNA*	Filamin A
*RETN*	Resistin
C/EBP	CCAAT/enhancer binding protein
FAS	Fatty acid synthase
HSL	Hormone-sensitive lipase
*ATGL*	Adipose triglyceride lipase
*TNF-α*	Tumor necrosis factor-α
