# Supplementary material for: Comparative Proteome Analysis Reveals Lipid Metabolism-Related Protein Networks in Response to Rump Fat Mobilization
Source: Int J Mol Sci. 2018 Aug 28;19(9):2556. doi: 10.3390/ijms19092556 (PMC6164786; doi:10.3390/ijms19092556)

Comparative Proteome Analysis Reveals Lipid Metabolism-Related Protein Networks in Response to Rump Fat Mobilization

Juanjuan Wang [1,2,†], Mengsi Xu [2,†], Xinhua Wang [2], Jinquan Yang [2], Lei Gao [2], Yan Zhang [3], Xin Huang [2], Mengli Han [2], Rui Gao [4,*] and Shangquan Gan [2,*]

[1] College of Animal Science and Technology, Shihezi University, Shihezi, 832000, China; wangjuanjuand10@163.com

[2] State Key Laboratory of Sheep Genetic Improvement and Healthy Production, Xinjiang Academy of Agricultural and Reclamation Sciences, Shihezi, 832000, China; xumengsi100@163.com (M.X.); wangxinhua5751@163.com (X.W.); xssxmjq@126.com (J.Y.); w.n007@163.com (L.G.); ahx512@163.com (X.H.); hanmenglimm@163.com (M.H.)

[3] Laboratory of Epigenetics, Beijing Institute of Biotechnology, Beijing, 100071, China; zany1983@gmail.com

[4] The Key Laboratory of Xinjiang Endemic & Ethnic Diseases and Department of Biochemistry, Shihezi University School of Medicine, Shihezi, 832000, China

* Correspondence: gaorui@shzu.edu.cn (R.G.); shangquangan@shzu.edu.cn (S.G.); Tel.: +86 (0)993 2863830 (R.G.); Tel.: +86 (0)993 6683739 (S.G.)

† These authors contributed equally to this work.

Running title: Proteins expression in fat deposition and mobilization

Keywords: Altay sheep; rump fat; proteomic; persistent starvation; Isobaric tags for relative or absolute quantitation (iTRAQ)

Figure S1. Establishment of animal models and the iTRAQ experiment

(A) Altay sheep models were established under conditions of progressive fasting, and adipose tissue collected before and after persistent starvation for iTRAQ analysis. (B) Schematic of the experimental design based on iTRAQ labeling combined with 2D LC-MS/MS analysis of FF and PS groups.

Figure S1. Establishment of animal models and the iTRAQ experiment

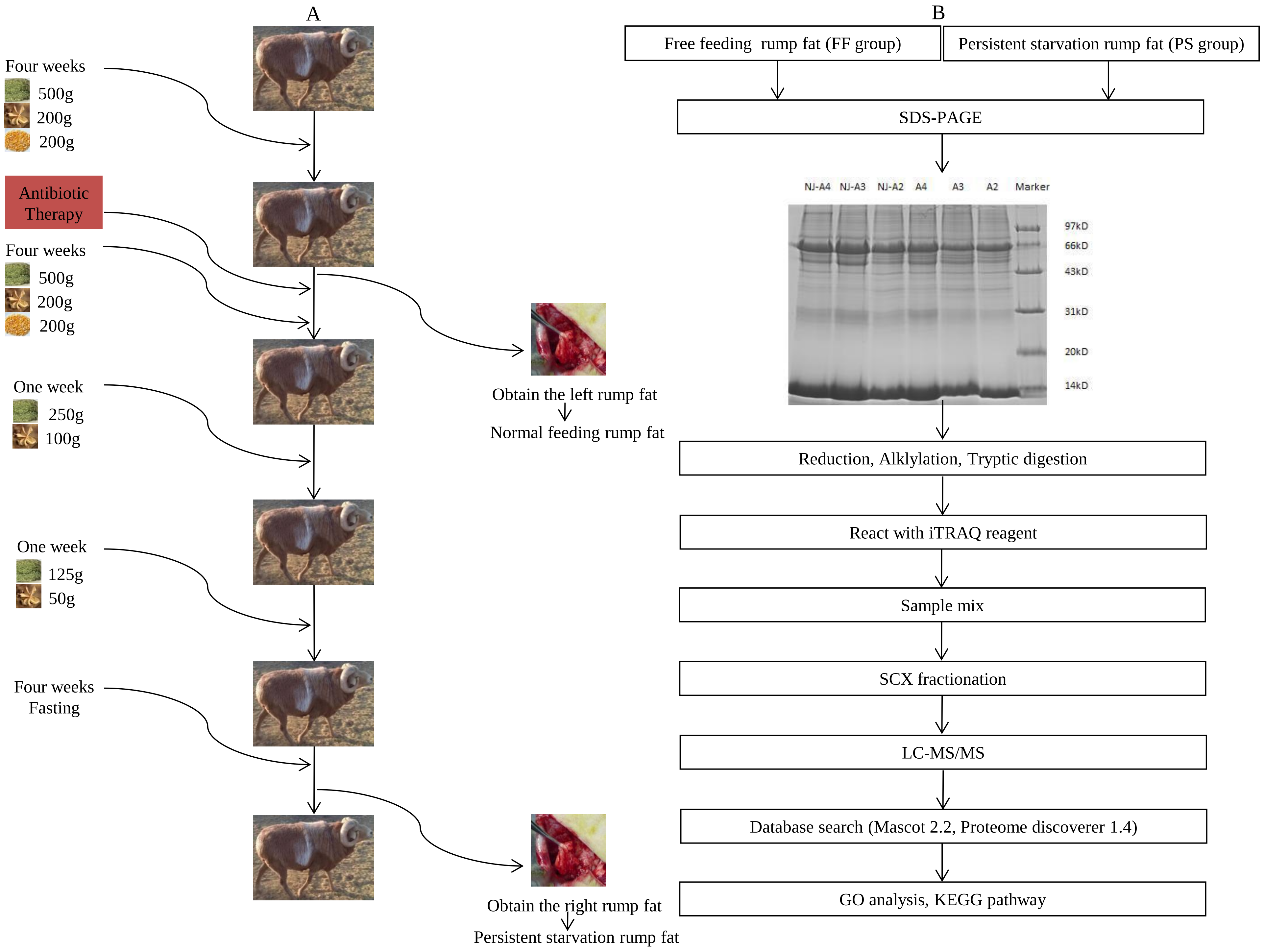

Supplement: Supplementary file 1 [file ijms-19-02556-s001.pdf]
